# The impact of implementing a standardized protocol for labor induction on obstetric disparities: Secondary analysis of a type I hybrid effectiveness‐implementation trial

**DOI:** 10.1002/pmf2.70077

**Published:** 2025-07-18

**Authors:** Rebecca F. Hamm, Sunni L. Mumford, Markolline Forkpa, Nicholas J. Seewald, Rinad S. Beidas, Sindhu K. Srinivas, Samuel Parry, Lisa D. Levine

**Affiliations:** ^1^ Department of Obstetrics & Gynecology University of Pennsylvania Perelman School of Medicine Philadelphia Pennsylvania USA; ^2^ Leonard Davis Institute of Health Economics Perelman School of Medicine University of Pennsylvania Philadelphia USA; ^3^ Department of Biostatistics Epidemiology and Informatics University of Pennsylvania Perelman School of Medicine Philadelphia Pennsylvania USA; ^4^ Department of Medical Social Sciences Feinberg School of Medicine Northwestern University Chicago Illinois USA

**Keywords:** cesarean delivery, health equity, implementation science, labor induction, maternal morbidity, obstetric disparities, standardization

## Abstract

**Introduction:**

Prior retrospective data demonstrated that standardization of labor induction may reduce racial disparities in cesarean delivery and morbidity. Here, we aimed to determine the impact of prospectively implementing an induction protocol on racially disparate outcomes.

**Methods:**

This was a planned secondary analysis of a type I hybrid effectiveness‐implementation trial comparing 2 years before (PRE) and 2 years after (POST) implementation of a standardized induction protocol at two labor units (2018 to 2022). The protocol had eight components and recommended active induction management, frequent cervical exams, and amniotomy by first exam ≥4 cm. All singleton pregnancies ≥37 weeks with intact membranes requiring cervical ripening were eligible; prior cesarean delivery was excluded. Data were collected via individual chart review. This analysis included only those with self‐identified race, divided into Black, Indigenous, People of Color (BIPOC), and white. Poisson regression with interaction terms evaluated the protocol's impact on disparities in cesarean delivery and morbidity. Fidelity to the protocol was defined as adherence to ≥75% of the eight protocol components.

**Results:**

A total of 8386 patients were included (PRE = 4167; POST = 4219); 59.3% were identified as BIPOC. BIPOC patients differed in delivery site, insurance, body mass index, parity, age, diagnosis of diabetes and hypertension, gestational age, and induction indication. BIPOC patients were more likely to undergo cesarean in the PRE (aRR 1.36[1.18–1.58]) period, and remained more likely to undergo cesarean POST‐implementation (aRR 1.55[1.33–1.70]), even when controlling for differences between groups. Similarly, maternal morbidity was greater among BIPOC patients PRE‐implementation (aRR 1.25[1.07–1.46]) and remained greater among BIPOC patients POST‐implementation (aRR 1.34[1.14–1.58]). There was no difference by race/ethnic group in neonatal morbidity in either PRE or POST. Finally, the protocol was implemented similarly by BIPOC versus white.

**Conclusion:**

Despite uniform implementation of a standardized induction protocol across race/ethnic groups, this intervention did not mitigate observed racial disparities in cesarean or maternal morbidity.

## 1 INTRODUCTION

There are significant, unacceptable disparities between those who identify as Black, Indigenous, and People of Color (BIPOC) and those who do not in the United States (US) in regard to birth outcomes [1, 2, 3]. For example, Black women and birthing people have more than 10% increased odds of cesarean delivery than non‐Hispanic White women, even when accounting for sociodemographic and clinical differences [4]. Prior work has examined interventions during the perinatal periods designed to improve racially disparate outcomes [5, 6]. These studies have shown improvement in care delivery, but have not necessarily proven effective in reducing morbidity. Thus, innovative interventions are needed to reduce obstetric racial disparities.

The utilization of protocols to standardize care has decreased adverse outcomes in various clinical fields [7, 8, 9, 10, 11, 12, 13, 14]. In addition, care standardization can minimize variation in medical decision‐making by race, thereby decreasing the impact of implicit bias [15]. With wide variation by site and by clinician across the US, labor induction management practices are an ideal target for standardization. Components of labor induction decision‐making, such as frequency of cervical examinations, utilization of artificial membrane rupture, and thresholds for cesarean, differ significantly across and within centers. Labor induction, which makes up 30% of all deliveries in the US, occurs in almost 1.2 million US women annually [16, 17]. Labor induction is associated with a cesarean delivery rate of around 25% nationally, thereby accounting for approximately 250,000 yearly US cesareans, making induction a critical target for interventions attempting to reduce cesarean rates [18]. In addition, with a large clinical trial supporting elective induction [19], induction rates are likely to continue to climb in coming years [20, 21].

Prior work retrospectively evaluated the hypothesis that standardizing labor induction practices can reduce racial disparities in obstetrics [22, 23]. Cesarean and maternal/neonatal morbidity were compared between women in a randomized trial of women undergoing induction with a standardized protocol versus a concurrent observational cohort of women being induced at clinician discretion. When stratified by race, there was a significant reduction in risk of cesarean for Black women in the protocol group compared to the observational group, while there was no such statistical difference for non‐Black women. In that retrospective study, the standardized protocol was associated with a significant reduction in the racial disparity in cesarean rate [22]. For this work, we aimed to determine the impact of prospectively implementing a standardized protocol for labor induction on racially disparate obstetric outcomes.

## 2 MATERIALS AND METHODS

This is a planned secondary analysis of a prospective cohort study using a pre‐ (PRE) and post‐ (POST) study design with implementation of a standardized labor induction protocol at two sites. The goal of the primary study was to determine the effectiveness of a standardized labor induction protocol on obstetric outcomes overall, while simultaneously evaluating implementation outcomes (a type I hybrid effectiveness‐implementation study) [24, 25]. The specifics of the utilized labor induction protocol (Figure S1), implementation strategies utilized to impact clinician behavior change, study methods, and outcomes of the primary study are described in prior work [25]. Briefly, the protocol included multiple components and recommended active management of labor induction, including frequent cervical examinations, amniotomy if cervical exam ≥4 cm, and interventions for labor dystocia. The goal of this secondary analysis was to evaluate the impact of implementing a standardized protocol for labor induction on racial disparities in obstetric outcomes including cesarean delivery, maternal morbidity, and neonatal morbidity.

Implementation of a labor induction protocol occurred in a stepped approach at two separate hospitals within the [hospital system redacted] from 2021 to 2022 (Figure S2). Both sites are urban hospitals with busy obstetrical services and racially diverse populations.

The project was approved by the University of Pennsylvania institutional review board as quality improvement with a waiver of informed consent. Strengthening the Reporting of Observational Studies in Epidemiology (STROBE) reporting guidelines were followed in the writing of this report [26].

### 2.1 Study population

In the primary study, pregnant patients were included in this study if they were undergoing a term (≥37 weeks) labor induction for any indication and met the following inclusion criteria: singleton gestation in cephalic presentation, intact membranes, and determined to require cervical ripening by their clinician. Both nulliparous and multiparous patients were included. Patients were excluded from the study if they had a prior cesarean delivery. For the purposes of this secondary analysis, those without self‐identified race and ethnicity recorded in the electronic health record were excluded, and patients were grouped as Black, Indigenous, People of Color (BIPOC) versus non‐Hispanic white. BIPOC included patients identifying as Black, Asian, Native Hawaiian/Pacific Islander, American Indian/Alaskan, or multiple racial groups, as well as those identifying as of Latinx ethnicity.

### 2.2 Outcomes

Demographic, labor course, and delivery data were collected on all patients meeting our criteria during the study period via individual chart review from the electronic health record. The primary outcome was cesarean delivery for any indication. Secondary maternal outcomes included: time to delivery (defined as time from start of induction to delivery in hours), clinical chorioamnionitis, postpartum hemorrhage (estimated blood loss ≥1000 cc for any mode of delivery), and composite maternal morbidity (defined as ≥1 of the following: endometritis, blood transfusion, wound infection or separation requiring intervention, venous thromboembolism, hysterectomy, intensive care unit admission, readmission within 30 days, or death). Secondary neonatal outcomes include composite neonatal morbidity (defined as ≥ one of the following: severe respiratory distress, culture‐proven sepsis requiring antibiotic therapy, neonatal hypoxic‐ischemic encephalopathy, intraventricular hemorrhage, or neonatal death) and neonatal intensive care unit (NICU) admission > 48 h. Severe respiratory distress was defined as intubation and/or additional oxygen support beyond nasal cannula (i.e., continuous positive airway pressure) required outside of the delivery room.

For purposes of fidelity, we selected eight components of the induction protocol (Figure S1) that could be evaluated objectively in chart review. Fidelity was defined as adherence to each of the eight specific protocol components (defined in Table 3) and was also assessed as adherence to ≥75% of the eight components for which an induction was eligible. For example, if an induction were only eligible for six components, adherence would be to ≥5 of those six components. If a patient underwent cesarean prior to reaching active labor, they would not be eligible for adherence to the three components specific to active labor.

### 2.3 Sample size

The baseline primary cesarean rate for patients undergoing labor induction at our institution was 33%. In order to detect a reduction to 28%, which was the primary cesarean rate seen in preliminary data for those utilizing an induction protocol [23], at a two‐sided alpha of 0.05 and power of 80%, we required 1330 patients in each of the PRE and POST groups in our primary study. To evaluate this prespecified sub‐aim and demonstrate that same reduction in cesarean delivery specifically among BIPOC patients, we accounted for the fact that ∼50% of our patient population identified as BIPOC, and thus required at least 2660 total patients in each of the PRE and POST groups. A total of 8800 deliveries occur per year among the included units, with approximately 18% of them meeting our inclusion and exclusion criteria [27]. Thus, a study period of 2 years PRE and 2 years POST data (3168 patients in each of the PRE and POST groups) was chosen to achieve the desired sample size for both the primary and secondary aims.

### 2.4 Analysis

Patients were grouped from both sites into PRE and POST implementation arms, which were further divided into BIPOC versus non‐Hispanic white. Comparisons were then made within the PRE as well as the POST implementation groups by race/ethnicity group. All continuous variables were assessed for distribution, and bivariate comparisons of demographic and pre‐induction clinical characteristics by race/ethnic group in the PRE‐ and POST‐implementation groups were performed with Fisher's exact tests for variables where any cell *n* ≤ 5, and *χ* ^2^ tests for all other categorical variables. Unpaired, 2‐tailed *t*‐tests were performed for parametric continuous variables, and Wilcoxon rank sum tests for nonparametric continuous variables. Unadjusted analyses for the primary and secondary clinical outcomes, as well as protocol fidelity, were performed in the same manner.

To perform adjusted analyses for clinical outcomes, variables associated with both the exposure (BIPOC vs. white in either the PRE or POST implementation groups) and the outcome independently at a *p* < 0.20 were assessed in modeling. Thus, delivery site, insurance status, body mass index (BMI), parity, maternal age, diagnosis of diabetes (pregestational or gestational), history of chronic hypertension, gestational age at delivery, and indication for induction were included. Modified Poisson regression modeling with robust error variance was used to estimate associations for binary outcomes while adjusting for all confounders, while Cox proportional hazards modeling was utilized for time‐to‐event outcomes, censoring for cesarean when evaluating length of labor. Linearity assumptions for Poisson regression modeling and proportional hazards assumptions for Cox modeling were assessed and deemed appropriate for each outcome variable.

To examine differences in the impact of implementing a standardized protocol for labor induction on clinical outcomes by race, the interaction between the pre‐ and post‐protocol implementation time periods and race/ethnic group was added to the above models. In this case, statistical significance identified with an interaction term would demonstrate a differential effect of the induction protocol implementation on the outcome by BIPOC versus white.

While some prior papers in this space utilized a division of race/ethnicity as Black versus non‐Black [22], BIPOC versus white was selected for the purposes of this paper given the growing recognition that obstetric outcomes differ significantly by these groupings [28], and a standardized labor induction protocol would have the opportunity to improve care for all patients of color. However, as prior work had retrospectively demonstrated improved disparities specifically non‐Hispanic Black patients as compared to non‐Hispanic white patients [22], a sensitivity analysis was performed comparing only those racial groups. Stratified analyses for the primary outcome were performed by parity. Statistical analyses were performed with Stata, version 15 (StataCorp LLC). All tests were 2 tailed, and *p* < 0.05 was considered statistically significant.

## 3 RESULTS

**FIGURE 1:**
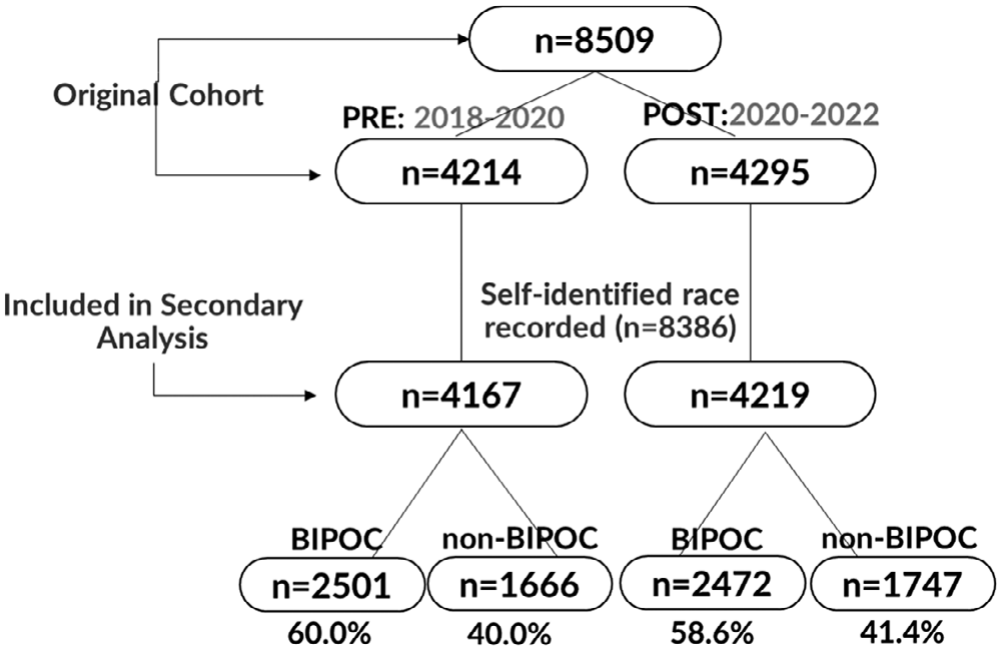
Study numbers.

Of the 8509 patients who met inclusion criteria in the parent study, 8386 (98.6%) had self‐reported race and ethnicity recorded and were included in this secondary analysis (PRE = 4167; POST = 4219). 60.0% and 58.6% identified as BIPOC in each of the PRE and POST implementation periods, respectively (Figure 1). Among BIPOC patients, 76.3% identified as Black, 11.7% Asian, 7.5% Hispanic‐white, and 4.4% other racial groups. Demographic and clinical characteristics comparing BIPOC and white groups within the PRE and POST implementation time periods are detailed in Table 1. BIPOC patients differed from white patients similarly in both the PRE and POST periods and this included differences in delivery site, insurance status, BMI, parity, maternal age, hypertension, gestational age, and indication for induction. Diagnosis of diabetes differed by race/ethnicity in the PRE‐implementation group, but was not statistically different in the POST‐implementation group.

**TABLE 1:** Demographic and clinical characteristics compared by BIPOC versus white race in each of the pre‐ and post‐implementation groups. This study sample includes all patients admitted for labor induction at either the Hospital of the University of Pennsylvania, Pennsylvania Hospital over the study period meeting inclusion and exclusion criteria for use of the standardized labor induction protocol who have a recorded self‐identified race.

	Pre (*n* = 4167)			Post (*n* = 4219)		
	BIPOC^a^ *n*(%) *N* = 2501	White^a^ *n*(%) *N* = 1666	*p* value	BIPOC^a^ *n*(%) *N* = 2472	White^a^ *n*(%) *N* = 1747	*p* value
Site						
*#1*	1614 (64.5)	483 (29.0)	<0.001	1572 (63.6)	556 (31.8)	<0.001
*#2*	887 (35.5)	1183 (71.0)		9000 (36.4)	1191 (68.2)	
Maternal age^b^	28.3 [23.6–32.9]	32.8 [30.4–35.7]	<0.001	28.8 [24.1–33.4]	33.3 [30.9–35.9]	<0.001
Insurance						
*Private*	961 (38.6)	1540 (92.6)	<0.001	943 (38.1)	1614 (92.4)	<0.001
*Medicaid/Medicare*	1529 (61.4)	123 (7.4)		1529 (61.9)	132 (7.6)	
Maternal BMI at last prenatal visit (mg/kg^2^)^b^	32.8 [28.3–38.6]	30.1 [27.2–34.2]	<0.001	33.3 [28.8‐38.9]	30.4 [27.4–34.6]	<0.001
Gestational or pregestational diabetes	262 (10.5)	131 (7.9)	0.005	277 (11.2)	165 (9.4)	0.07
Chronic hypertension	249 (10.0)	86 (5.2)	<0.001	270 (10.9)	128 (7.3)	<0.001
Nulliparity	1490 (59.6)	1241 (74.5)	<0.001	1426 (57.7)	1295 (74.1)	<0.00 1
Gestational age at delivery^b^	39.4 [38.4‐40.2]	39.6 [39.1‐40.5]	<0.001	39.4 [38.6‐40.2]	39.6 [38.9–40.4]	<0.001
Indication for induction			<0.001			<0.001
*Postdates/elective*	627 (25.1)	715 (42.9)		640 (25.9)	687 (39.3)	
*Maternal indications* ^c^	910 (36.4)	537 (32.2)		903 (36.5)	663 (38.0)	
*Fetal indications* ^d^	652 (26.1)	208 (12.5)		551 (22.3)	179 (10.2)	
*Other* ^e^	312 (12.5)	206 (12.4)		378 (15.3)	218 (12.5)	
Modified Bishop score^b^	2 [0‐3]	2 [0‐3]	0.72	2 [0‐3]	2 [0–3]	0.93

*Note*: Data are presented as *n*(%) unless otherwise stated.

^a^ Black, Indigenous, People of Color.

^b^ Median[IQR].

^c^ Examples include: chronic hypertension, gestational hypertension, preeclampsia, diabetes, renal disease, history of venous thromboembolism, cardiac disease or other chronic medical condition where induction was recommended.

^d^ Examples include: oligohydramnios, intrauterine growth restriction, abnormality on fetal testing.

^e^ Examples of “other” include: history of an intrauterine fetal demise, vaginal bleeding at term, cholestasis.

For the primary outcome, while unadjusted analyses did not differ, when adjusting for differences between groups in regression modeling, BIPOC patients had a higher risk of cesarean in both the PRE (aRR 1.36 95% CI [1.18–1.58]) and POST periods (aRR 1.55 95% CI [1.33–1.70]; Table 2), despite implementation of a standardized protocol for labor induction. This “unmasking” of associations in adjusted analyses were largely driven by differences in parity, supporting planned stratified analyses. BIPOC patients were also more likely to be diagnosed with both chorioamnionitis and postpartum hemorrhage in both the PRE and POST implementation periods once controlling for between‐race differences. Similarly, the risk of composite maternal morbidity was greater among BIPOC patients as compared to white patients in both the PRE (aRR 1.33 95% CI [1.13–1.56]) and POST (aRR 1.46 95% CI [1.25–1.72]) periods. Time to delivery was shorter for BIPOC patients compared to white patients in both the PRE and POST periods, but this finding was no longer significant in adjusted analyses. Finally, composite neonatal morbidity and NICU admission did not differ by race/ethnic group in this cohort in either the PRE or POST period, in unadjusted or adjusted analyses.

**TABLE 2:** Primary and secondary maternal and neonatal clinical effectiveness outcomes compared by BIPOC versus white race in each of the pre‐ and post‐implementation groups.

	Pre (n = 4167)				Post (*n* = 4219)				
	BIPOC^a^ *n*(%) *N* = 2501	White^a^ *n*(%) *N* = 1666	*p* value^b^	aRR [95%CI]^c^	BIPOC^a^ *n*(%) *N* = 2472	White^a^ *n*(%) *N* = 1747	*p* value^b^	aRR [95%CI]^c^	Interaction *p* ^d^
**Primary Outcome**									
Cesarean delivery	550 (22.0)	350 (21.0)	0.45	1.36 [1.18–1.58]	575 (23.3)	347 (19.9)	0.009	1.55 [1.33–1.70]	0.09
**Secondary Maternal Outcomes**									
Chorioamnionitis	295 (11.8)	179 (10.7)	0.30	1.27 [1.01–1.60]	277 (11.2)	154 (8.8)	0.01	1.34 [1.05–1.70]	0.27
Postpartum hemorrhage^e^	345 (14.1)	239 (14.6)	0.63	1.23 [1.00–1.51]	314 (12.9)	212 (12.4)	0.61	1.44 [1.17–1.78]	0.94
Composite maternal morbidity^f^	235 (9.4)	155 (9.3)	0.92	1.33 [1.13–1.56]	157 (6.4)	114 (6.5)	0.82	1.46 [1.25–1.72]	0.07
Time to delivery (h)^g^, ^h^	16.0 [10.9‐23.9]	19.2 [13.5–26.8]	<0.001	0.99 [0.91–1.07]^i^	16.8 [11.0‐24.9]	20.0 [13.4‐28.3]	<0.001	0.93 [0.86‐1.00]^i^	0.25
**Secondary Neonatal Outcomes**									
Composite neonatal morbidity^j^	62 (2.5)	38 (2.3)	0.68	1.14 [0.76–1.72]	64 (2.6)	63 (3.6)	0.06	0.75 [0.53–1.07]	0.16
NICU admission > 48 h^k^	125 (5.0)	68 (4.1)	0.17	1.17 [0.77–1.61]	147 (5.6)	100 (5.7)	0.76	0.99 [0.72–1.36]	0.75

*Note*: Data are presented as *n*(%) unless otherwise stated.

^a^ Black, Indigenous, People of Color.

^b^ *p* value for unadjusted analyses.

^c^ Adjusted risk ratio controlling for delivery site, insurance, BMI, parity, maternal age, diagnosis of diabetes and hypertension, gestational age, and IOL indication.

^d^ Evaluates the differential impact of the protocol on a given outcome by race.

^e^ ≥1 100cc.

^f^ ≥1 of the following: endometritis, blood transfusion, wound infection or separation (requiring intervention), venous thromboembolism, hysterectomy, intensive care unit admission, readmission, and death within 30 days of delivery.

^g^ Defined as time from start of induction to delivery.

^h^ Median[IQR].

^i^ Adjusted Hazard Ratio (HR) with 95% CI, censored for cesarean where applicable.

^j^ Defined as ≥1 of the following: severe respiratory distress, culture‐proven sepsis requiring antibiotic therapy, neonatal hypoxic‐ischemic encephalopathy, intraventricular hemorrhage, or neonatal death.

^k^ Neonatal intensive care unit.

When examining the impact of implementing the standardized protocol for labor induction on clinical outcomes by BIPOC versus white, the interaction between PRE‐ and POST‐implementation time‐period and race/ethnic grouping were evaluated. Importantly, there was no differential effect of the standardized protocol on cesarean delivery or other maternal outcomes by BIPOC versus white. In other words, implementation of the protocol did not impact the racial/ethnic disparity (the degree of difference by race/ethnic grouping) between cesarean delivery and outcomes as indicated by *p* values > 0.05 for all interaction terms across analyses (Table 2).

Table 3 includes the eight protocol components that were assessed for fidelity to the standardized labor induction protocol, compared by BIPOC versus white in each of our PRE‐ and POST‐implementation groups. For component #1, “if cervical ripening balloon is utilized, if remains in place at 12 h after placement, remove it and initiate/continue oxytocin,” there was no difference by BIPOC versus white in component fidelity in the PRE implementation period, but fidelity to this component was higher for BIPOC patients in the POST period. While this difference was not significant in adjusted analyses, a significant interaction term (*p* = 0.04) indicates a differential impact of the protocol on fidelity to this component by race/ethnic group. Unadjusted analyses indicated differences in utilization of protocol components #3, #4, and #6 by BIPOC versus white across both time periods, but there were no associations between utilization of these components and race/ethnic group in either the PRE or POST implementation periods in adjusted analysis. There were no significant interactions between the protocol implementation and BIPOC versus white in any components other than component #1.

**TABLE 3:** Fidelity to eight individual components of the labor induction protocol compared by BIPOC versus white race in each of the pre‐ and post‐implementation groups.

	Pre (*n* = 4167)				Post (*n* = 4219)				
	BIPOC^a^ *n*(%) *N* = 2501	White^a^ *n*(%) *N* = 1666	*p* value^b^	aRR [95%CI]^c^	BIPOC^a^ *n*(%) *N* = 2472	White^a^ *n*(%) *N* = 1747	*p* value^b^	aRR [95%CI]^c^	Interaction *p* ^d^
Protocol Recommendation									
1. If cervical ripening balloon is utilized, if remains in place at 12 h after placement, remove it and initiate/continue oxytocin.	2065 (95.5)	1306 (94.4)	0.16	1.15 [0.79–1.66]	2008 (97.3)	1340 (94.6)	<0.001	0.72 [0.47–1.12]	0.04
2. If misoprostol is utilized, it should only be repeated for up to a total of 6 doses and for no > 24 h. If remains in latent labor, initiate oxytocin.	2205 (99.6)	1463 (99.2)	0.16	1.33 [0.52–3.40]	2265 (99.5)	1593 (99.4)	0.74	1.56 [0.56–4.31]	0.54
3. If it has been more than 6 h since misoprostol placement (whether or not cervical ripening balloon is in place), and AROM not yet feasible with no window for another misoprostol, start oxytocin.^e^, ^f^	1626 (73.4)	861 (58.4)	<0.001	0.94 [0.82–1.08]	1670 (73.4)	1014 (63.3)	<0.001	1.01 [0.88–1.17]	0.28
4. Latent labor exams should be performed: At least every 3 h if misoprostol and/or Foley being used; At least every 4 h if oxytocin is being used.	1048 (41.9)	415 (24.9)	<0.001	1.01 [0.88–1.17]	1165 (47.1)	562 (32.2)	<0.001	1.01 [0.89–1.15]	0.14
5. If patient is ≥4 cm dilated and has intact membranes, recommend performing amniotomy if feasible.	1529 (61.9)	1040 (63.3)	0.36	0.95 [0.82–1.10]	1685 (68.8)	1207 (70.00	0.55	0.96 [0.85–1.10]	0.81
6. Exams should be performed every 1–2 h in active labor.^g^	1904 (89.1)	1222 (80.8)	<0.001	1.01 [0.81–1.25]	1880 (88.6)	1290 (82.1)	<0.001	1.14 [0.92–1.40]	0.43
7. If there are 2 exams in active labor 2 h apart with the same cervical dilation and membranes are already ruptured, but oxytocin has not yet been started, start oxytocin.	6/20 (30.0)	3/11 (27.3)	0.87	1.81 [0.10–32.41]	8/25 (32)	4/10 (40)	0.65	1.79 [0.20–15.67]	0.84
8. If there are 2 exams in active labor 2 h apart with the same cervical dilation and membranes are already ruptured, with oxytocin already begun, place an IUPC.^h^	36/97 (37.1)	23/90 (25.6)	0.09	1.41 [0.94–2.11]	61 (56.5)	35/77 (45.5)	0.14	1.13 [0.82–1.55]	0.26

^a^ Black, Indigenous, People of Color.

^b^ p‐value for unadjusted analyses.

^c^ Adjusted risk ratio controlling for delivery site, insurance, BMI, parity, maternal age, diagnosis of diabetes and hypertension, gestational age, and IOL indication.

^d^ Evaluates the differential impact of the protocol on a given outcome by race.

^e^ AROM = Artificial Rupture of Membranes.

^f^ The measure was no longer assessed once either oxytocin was initiated or AROM occurred, as this was determined to be the completion of cervical ripening.

^g^ Among those who reached active labor ≥6 cm dilation.

^h^ IUPC = intrauterine pressure catheter.

Finally, there was no differential impact of protocol implementation by race/ethnic group in inductions adherent to ≥75% of the eight protocol components for which a patient was eligible (interaction *p* = 0.15).

Sensitivity analyses were performed for primary and secondary outcomes comparing only non‐Hispanic Black and non‐Hispanic white patients (Table S1). Similar to the primary analysis, when adjusting for differences between groups in regression modeling, Black patients had a higher risk of cesarean in both the PRE and POST periods (PRE aRR 1.72 [1.35–2.20]; POST aRR 1.88 [1.47–2.39]), despite implementation of a standardized protocol for labor induction. Notably, in this analysis, there was a differential effect of the standardized protocol on cesarean delivery by Black versus white racial group.

Stratified analysis by parity demonstrated similar findings for nulliparous patients, where there was a higher cesarean rate for BIPOC nulliparas in both the PRE and POST periods (PRE aRR 1.64 [1.25–2.15]; POST aRR 1.84 [1.42–2.40]), with an interaction term also representative of a statistically significant impact of the protocol on the degree of difference in cesarean delivery by race (Table S2; interaction *p* = 0.004). There was no statistically significant impact of the protocol on cesarean for multiparous patients.

## 4 DISCUSSION

In this planned, secondary analysis of a type I hybrid effectiveness‐implementation trial, we evaluated the prospective impact of standardizing management of labor induction on racial disparities in obstetric outcomes. Despite implementation of a standardized protocol across racial groups, BIPOC patients remained more likely to undergo cesarean and experience maternal morbidity in both the PRE‐ and POST‐implementation groups. There was no statistically significant difference by BIPOC versus white in neonatal morbidity in either PRE‐ or POST‐implementation. Implementation of the protocol had no differential impact by race/ethnic grouping on cesarean or maternal or neonatal morbidity. There was no difference in protocol fidelity post‐implementation across racial groups, both overall and by individual components, save for one component (Foley balloon utilization), which was better implemented among the BIPOC population as compared to the white population.

When the analysis was limited to only Black versus white patients, the protocol appeared to have a statistically significant effect on cesarean by race. In addition, this finding was also seen among BIPOC versus white patients when the analysis was limited to nulliparous patients. However, for both of these comparisons, the relative risk of cesarean by race remained similar in both the PRE and POST groups, with low cesarean rates for all groups across the entire study period.

Strengths of our study include the large, diverse sample size of over 8300 inductions across two sites, among which more than half of patients identified as BIPOC. Given this was a planned secondary analysis, we were powered to evaluate the impact of prospectively standardizing induction management on racial disparities in critical obstetric outcome. Our study, however, is limited by its pre‐post implementation design, susceptible to unmeasured bias and secular trends. Furthermore, due to the team‐ and shift‐based care model of the included delivery sites, we were unable to control for individual clinicians and their impact on disparities in these outcomes. Second, it is plausible that although implementation occurred similarly across racial groups, and adherence to many evidence‐based induction management strategies increased overall in the primary study [25], it is plausible that it did not occur successfully enough across any race/ethnic group to drive reductions in obstetric disparities. For example, adherence to the recommendation to perform regular cervical examinations during latent labor rose close to seven percentage points, but remained below 70% in both racial groups even after implementation of the protocol. It is possible that with greater fidelity to more of the evidence‐based practices, or to specific individual evidence‐based practices, greater clinical changes in racial disparities could have been observed.

Race is also a complex social construct, and lack of granularity in understanding race and ethnicity through self‐recorded mechanisms in the health record makes evaluating the true exposure, systemic racism, a significant challenge. Protocol implementation is also dependent not just on clinician behavior change, but also on patient acceptability of each component. Mixed‐methods studies evaluating patient perspectives on the protocol are underway. Finally, obstetric disparities are multifactorial, rooted in social drivers of health that highly influence health status coming into pregnancy and delivery. As a result, an intervention at the point of labor management may be too late in the care continuum or insufficient to drive meaningful change.

Prior, smaller‐scale retrospective work out of our group demonstrated that utilization of a standardized protocol for labor induction was associated with reduced racial disparities in cesarean delivery rate and neonatal morbidity [22]. Contrary to that, however, another retrospective study from our institution found that while BIPOC nulliparas undergoing an induction were 40% more likely to undergo cesarean during induction than white patients, there were no identifiable differences by race in labor induction management prior to the decision for cesarean [28]. Specifically, there were no differences in choice of cervical ripening agent, cervical dilation at or time to amniotomy, use and maximum dose of oxytocin, or dilation at cesarean. These data suggest there may be less room for standardized labor induction management to impact obstetric disparities, supporting the results of the current study.

Other pre–post implementation work outside of our institution evaluating the impact of standardizing labor induction on racial disparities in outcomes also failed to show significant impacts [29]. Triebwasser et al. performed a sensitivity analysis in the context of a hospital‐wide quality improvement initiative comparing outcomes among non‐Hispanic Black and white patients before and after standardizing induction, which demonstrated no differences. However, limitations were noted in the small sample size of non‐Hispanic Black patients, and the fact that no disparities were noted by race even in their PRE‐implementation cohort.

Despite the promise of care standardization for racial disparity reduction in health, in context to the current study, data are insufficient to state that implementation of a standardized protocol for labor induction would significantly impact racially disparate obstetric outcomes. Yet, this does not undermine the benefits seen from protocol implementation for populations overall [25]. Specifically, in the overall study [25], implementation of standardized protocol for labor induction was associated with tangible improvements in active induction management, and improvement in maternal morbidity, without a change in the overall cesarean delivery rate, labor length, or neonatal morbidity. In addition, while positive outliers exist [30], at labor units where differences in labor management are more prevalent, or baseline cesarean rates are higher, there remains potential for this intervention to impact disparities in those locations.

However, it is also critically important to continue to consider alternative interventions to reduce the profound racial disparities present in cesarean delivery and maternal morbidity. The findings from this study support the hypothesis that more promising interventions might target time points prior to presentation for labor, such as those improving social drivers of health and access to care, as well as interventions that target clinician bias in the decision for cesarean. Future work might determine more specifically where bias in cesarean decision‐making occurs to generate directed solutions, such as through an ethnography of how decisions are made for cesarean from the patient, family, and clinician perspectives.

## 5 CONCLUSION

Despite implementation of a standardized induction protocol across racial groups, this intervention did not mitigate observed racial disparities in cesarean delivery or maternal morbidity rates.

## AUTHOR CONTRIBUTIONS

R.F.H., L.D.L., S.P., S.K.S., and R.B. conceived of and designed this work. R.F.H. collected data. All authors assisted in data interpretation. R.F.H. drafted the manuscript, which was revised by all authors. All authors have approved the final manuscript.

## CONFLICT OF INTEREST STATEMENT

The authors declare no conflicts of interest.

## ETHICS APPROVAL

The project was approved by the University of Pennsylvania Institutional Review Board as quality improvement, and written informed consent was thereby waived.

## Supporting information



Supporting Information

Supporting Information

## Data Availability

The data that support the findings of this study are available from the corresponding author upon reasonable request.
