# Supplementary material for: The impact of implementing a standardized protocol for labor induction on obstetric disparities: Secondary analysis of a type I hybrid effectiveness‐implementation trial
Source: Pregnancy (Hoboken). 2025 Jul 18;1(4):e70077. doi: 10.1002/pmf2.70077 (PMC13344648; doi:10.1002/pmf2.70077)
Supplement: Supplementary file 1 [file PMF2-1-e70077-s001.docx]

Supplemental Figure 1:

Figure 1a: Standardized protocol for the management of latent labor. 1b: Standardized protocol for the management of active labor.

Supplemental Figure 2: Study Design
