# Supplementary material for: The impact of implementing a standardized protocol for labor induction on obstetric disparities: Secondary analysis of a type I hybrid effectiveness‐implementation trial
Source: Pregnancy (Hoboken). 2025 Jul 18;1(4):e70077. doi: 10.1002/pmf2.70077 (PMC13344648; doi:10.1002/pmf2.70077)
Supplement: Supplementary file 2 [file PMF2-1-e70077-s002.docx]

Supplemental Table 1: Sensitivity analysis for primary and secondary maternal and neonatal clinical effectiveness outcomes compared by Black versus white race in each of the pre- and post- implementation groups.

|  | **Pre (n=3581)** | | | | **Post (n=3628)** | | | |  |
| --- | --- | --- | --- | --- | --- | --- | --- | --- | --- |
|  | **Black**^a^ **n(%)**  **N=1915** | **White n(%)**  **N=1666** | **p-value** ^b^ | **aRR [95%CI]**^c^ | **Black**^a^ **n(%)**  **N=1881** | **White n(%)**  **N=1747** | **p-value** ^b^ | **aRR [95%CI]**^c^ | **Interaction p^d^** |
| **Primary Outcome:** Cesarean delivery | 404 (21.1) | 350 (21.0) | 0.95 | 1.72 [1.35-2.20] | 425 (22.6) | 347 (19.9) | 0.045 | 1.88 [1.47-2.39] | 0.015 |
|  | | | | | | | | | |
| Chorioamnionitis | 200 (10.4) | 179 (10.7) | 0.77 | 1.00 [0.73-1.37] | 198 (10.5) | 154 (8.8) | 0.08 | 1.22 [0.88-1.68] | 0.11 |
| Postpartum hemorrhage ^e^ | 246 (13.1) | 239 (14.6) | 0.20 | 1.26 [0.96-1.67] | 228 (12.3) | 212 (12.4) | 0.97 | 1.63 [1.23-2.17] | 0.16 |
| Composite maternal morbidity ^f^ | 177 (9.2) | 155 (9.3) | 0.95 | 1.17 [0.85-1.61] | 116 (6.2) | 114 (6.5) | 0.66 | 1.31 [0.90-1.91] | 0.90 |
| Time to delivery (hours)^g,h^ | 15.5 [10.5-23.3] | 19.2 [13.5-26.8] | <0.001 | 1.35 [1.12-1.64] | 16.3 [10.7-24.5] | 20.0 [13.4-28.3] | <0.001 | 1.17 [0.97-1.42] | 0.35 |
|  | | | | | | | | | |
| Composite neonatal morbidity ^j^ | 50 (2.6) | 38 (2.3) | 0.52 | 0.77 [0.41-1.45] | 53 (2.8) | 63 (3.6) | 0.18 | 0.82 [0.49-1.39] | 0.30 |
| NICU admission >48 hours ^k^ | 101 (5.3) | 68 (4.1) | 0.09 | 1.16 [0.75-1.80] | 118 (6.3) | 100 (5.7) | 0.49 | 1.05 [0.71-1.56] | 0.70 |
| Data are presented as n(%) unless otherwise stated^. a^ non-Hispanic Black ^b^ p-value for unadjusted analyses ^c^Adjusted risk ratio controlling for delivery site, insurance, BMI, parity, maternal age, diagnosis of diabetes and hypertension, gestational age, and IOL indication ^d^Evaluates the differential impact of the protocol on a given outcome by race ^e^  ≥1 100cc ^f^ ≥1 of the following: endometritis, blood transfusion, wound infection or separation (requiring intervention), venous thromboembolism, hysterectomy, intensive care unit admission, readmission, and death within 30 days of delivery ^g^defined as time from start of induction to delivery ^h^Median[IQR] ^i^ Adjusted Hazard Ratio (HR) with 95% CI, censored for cesarean where applicable ^j^defined as ≥1 of the following: severe respiratory distress, culture-proven sepsis requiring antibiotic therapy, neonatal hypoxic-ischemic encephalopathy, intraventricular hemorrhage, or neonatal death ^k^Neonatal intensive care unit | | | | | | | | | |

Supplemental Table 2: Stratified analysis by parity, comparing cesarean delivery by BIPOC versus white among pre- and post- implementation groups.

|  | **Pre (n=4167)** | | | | **Post (n=4219)** | | | |  |
| --- | --- | --- | --- | --- | --- | --- | --- | --- | --- |
|  | **BIPOC**^a^ **n(%)**  **N=2501** | **White**^a^ **n(%)**  **N=1666** | **p-value** ^b^ | **aRR [95%CI]**^c^ | **BIPOC**^a^ **n(%)**  **N=2472** | **White** ^a^ **n(%)**  **N=1747** | **p-value** ^b^ | **aRR [95%CI]**^c^ | **Interaction p^d^** |
| **Nulliparous patients only:** Cesarean delivery | 466 (31.3) | 325 (26.2) | 0.004 | 1.64 [1.25-2.15] | 499 (35.0) | 327 (25.3) | <0.001 | 1.84 [1.42-2.40] | 0.004 |
| **Multiparous patients only:** Cesarean delivery | 84 (8.3) | 25 (5.9) | 0.11 | 1.92 [1.02-3.60] | 76 (7.3) | 20 (4.4) | 0.04 | 1.66 [0.86-3.20] | 0.89 |
| ^. a^ Black, Indigenous, People of Color ^b^ p-value for unadjusted analyses ^c^Adjusted risk ratio controlling for delivery site, insurance, BMI, parity, maternal age, diagnosis of diabetes and hypertension, gestational age, and IOL indication ^d^Evaluates the differential impact of the protocol on a given outcome by race | | | | | | | | | |
